# Ejaculatory duct cyst causing bilateral obstruction and subsequent infertility: A rare case report from Indonesia^☆^^☆^

**DOI:** 10.1016/j.radcr.2022.01.011

**Published:** 2022-01-17

**Authors:** Fandy Wicaksono, Yudhistira Pradnyan Kloping, Fikri Rizaldi, Doddy Moesbadianto Soebadi

**Affiliations:** a Department of Urology, Faculty of Medicine, Universitas Airlangga, Surabaya, East Java, Indonesia; b Dr. Soetomo General-Academic Hospital, Surabaya, East Java, Indonesia; c Rumah Sakit Universitas Airlangga Teaching Hospital, Surabaya, East Java, Indonesia

**Keywords:** Ejaculatory duct cyst, Ejaculatory duct obstruction, Male infertility, Case report

## Abstract

An ejaculatory duct cyst is a rare and challenging problem for a urologist. Diagnosing these cases may be difficult as prior case reports showed that the condition has various clinical presentations from asymptomatic to major complications, such as recurrent urinary tract infections and infertility. We aimed to report a 26-year-old married man with seemingly unknown infertility, which was confirmed to be due to an ejaculatory duct cyst obstructing both ejaculatory ducts.

## Introduction

Pelvic cysts in men are mostly benign and asymptomatic; thus, they tend to be discovered as incidental findings during imaging examinations of other conditions. In recent years, the use of pelvic MRI for genitourinary malignancy has increased, which consequently also increased the depiction of male pelvic cysts [1]. Cysts of the male pelvis can be divided into Müllerian cysts, seminal vesicle cysts, ejaculatory duct cysts, and cysts of the prostate based on their anatomical origins [2]. Diagnosing these cases could be difficult as prior reports showed that the condition has various clinical presentations, including pain in the lower abdominal region, recurrent urinary tract infections, painful ejaculation, hematospermia, and infertility [3]. Pelvic cysts originating from the Mullerian and Wolffian ducts are common, however, Ejaculatory duct cysts are very rare, with only a few reports in the literature [4,5]. Whether congenital or acquired, it may obstruct the ejaculatory duct and cause ejaculatory duct obstruction (EDO), which accounts for almost 5% of the male infertility [6]. A person who failed to conceive after a year of regular unprotected sexual intercourse is considered to be infertile. One of the main etiopathology of male infertility is secondary, such as varicocele and obstruction of the male genital tract, hypogonadism, cryptorchidism, and other endocrinopathies [7]. Male genital tract obstruction, including EDO, is reversible as long as it is identified and treated properly as spermatogenesis is well preserved even when there is obstruction [8]. Therefore, a proper understanding of the condition, including how to diagnose and treat the condition is necessary to help the patient and his spouse fulfill their need as a married couple. We aimed to report a case of a 26-year-old married man with an ejaculatory duct cyst causing bilateral EDO and subsequent infertility.

## Case presentation

An otherwise healthy 26-year-old male came with a complaint of infertility. He also complained of dry orgasm for 8 years. During intercourse, the patient could attain an adequate erection with a normal sensation of orgasm but could not ejaculate fluid. The patient had regular intercourse 3 times per week with his current partner and had normal penetration, without any pain during intercourse and pain during the climax, with an average climax duration of 10 minutes after penetration. The patient had normal urination without hematospermia and any other systemic symptoms. He was previously married to a different partner for 6 months without any children. His current wife has never been to a gynecologist. There was no history of spinal trauma, testicular trauma, or surgery. From the family history, the patient reported that his uncle had a history of infertility. Furthermore, there were no families with malignancies or congenital defects. The patient's vital signs were normal throughout the physical examination. There were no abnormalities in the chest or abdomen, however, the patient had an obese posture. The genitalia external examination showed a circumcised penis with a normal appearance of the glans. On scrotal examination, the left scrotum appears to be larger than the right, with normal palpable testicles, normal consistency, and no tenderness. The epididymis was noticeably larger on both sides with palpable vas deferens, no varicocele, and positive transillumination tests on both sides of the testicles. There were no abnormal findings based on the digital rectal examination. His laboratory results were within normal limits.

## Investigations/imaging findings

**Fig. 1:**
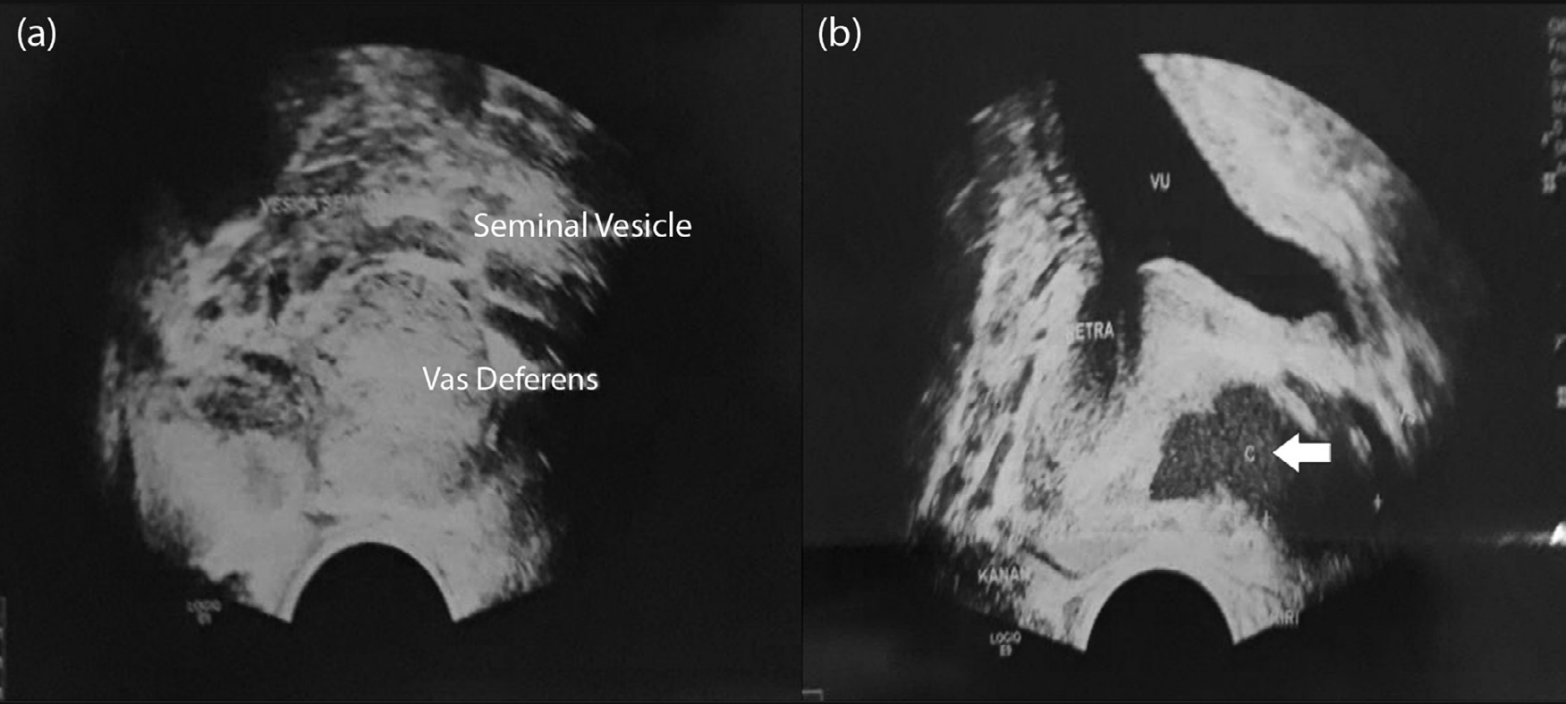
(A, B). TRUS image showing the dilation of vas deferens and seminal vesicles with a cystic lesion between the two structures, as shown by the white arrows.

**Fig. 2:**
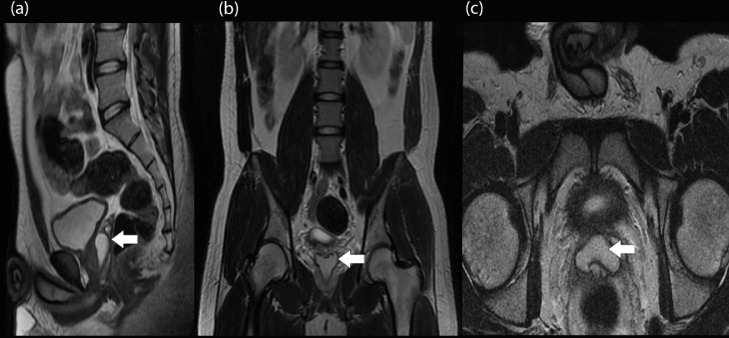
Contrast-Enhanced abdominal MRI showing a hyperintense lesion on TW1, indicating a cyst from the (A) sagittal, (B) coronal, and (C) axial views, as shown by the white arrows.

Chest and Abdominal X-Ray images revealed no abnormalities. Transurethral ultrasound (TRUS) examination in Figure 1 showed that left vas deferens and seminal vesicles appeared to be dilated with a 3.2 × 1.29 cm cystic lesion between them. A contrast-enhanced abdominal MRI examination was performed afterward, as shown in Figure 2. The images showed a hyperintense lesion on TW1 with a clear border, irregular edges, with a size of 2.3 × 3.2 × 2.9 cm without contrast enhancement on the openings of the ejaculatory duct. This finding suggested a high protein content cyst in the opening of the ejaculatory duct that connects to the right and left seminal vesicles and a bilateral hydrocele.

### Differential diagnosis

The patient was diagnosed with an ejaculatory duct cyst based on the MRI and USG results.

### Treatment

**Fig. 3:**
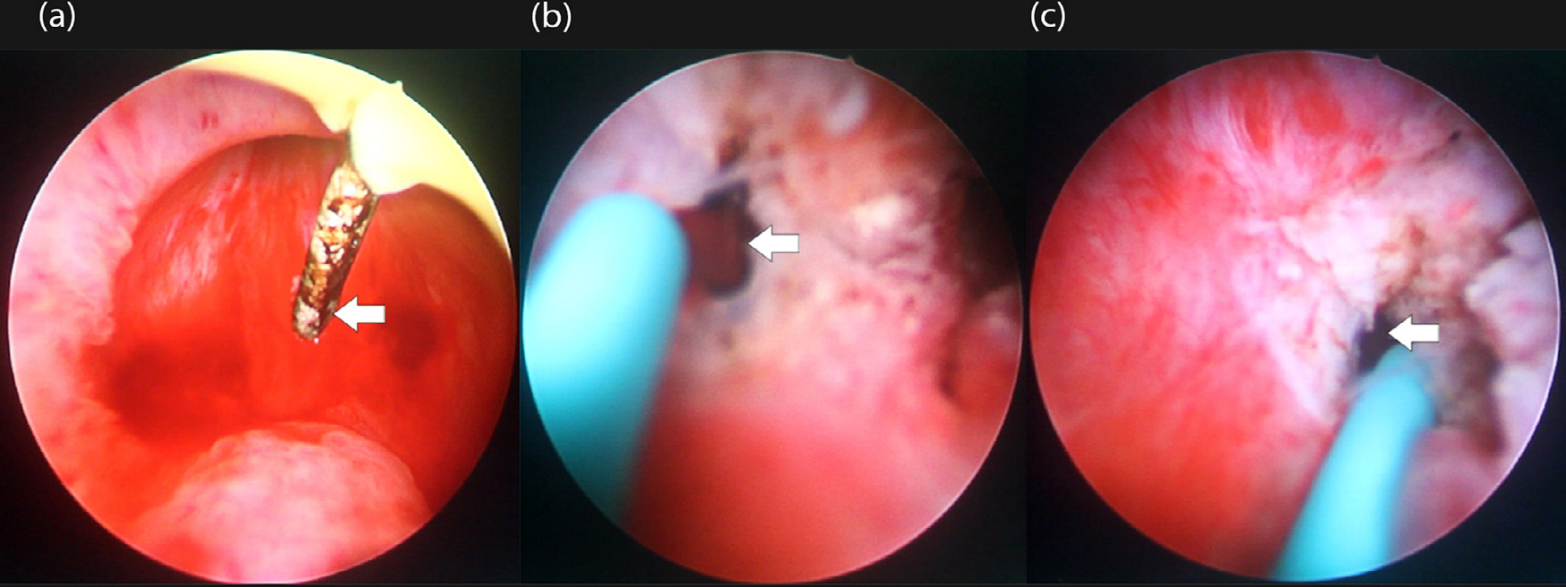
TURED procedure showing the cyst (A) incision using Collin's knife and (B, C) aspiration via the access made by the incision, as shown by the white arrows.

The patient was planned for transurethral resection of the ejaculatory duct (TURED) with regional anesthesia. The urinary tract examination was performed using a 0.30 optic cystourethroscopy, revealing a normal urethra, bladder neck, and bladder. The verumontanum showed protrusions and hyperemia. The first step of the procedure involved dilating the urethra using a 27 Fr Benique dilator. Collins Knife was used to incise the mass, resulting in a yellowish liquid pouring from the verumontanum, as shown in Figure 3. To safely evacuate the content from the cyst, aspiration was performed using a 5 Fr ureteral catheter (UC), placed in the right and left ejaculatory ducts. Prior to the aspiration, active rinsing was performed to concentrate the fluid inside the cyst using 10 mL NaCl 0.9%. A total of 20 cc of blackish fluid from the right ejaculatory duct and 10 cc of brownish fluid from the left ejaculatory duct were obtained from the aspiration and further examined for a semen analysis. The specimen taken from the right ejaculatory duct was dark red, resulting in a pH of 7.2, an immotile sperm count of 0-3 per field of view, and 36.77 mmol fructose. The second specimen from the left ejaculatory duct was clear-reddish in color, resulting in a pH of 7.0, an immotile sperm count of 0-2 per field of view, and 3.23 mmol fructose. A 16 Fr urinary catheter was placed afterward. The procedure was considered successful as the patient did not show any perioperative complications and was discharged 2 days after the procedure.

### Outcome and follow-up

One week following the procedure, the patient came to the outpatient clinic without any complaints. One month after the procedure, sperm analysis showed an increasingly small amount of sperm content with a total of 10-20 motile sperm per field of view. Unfortunately, we weren't able to confirm the patient's wife's pregnancy status as the patient was lost to follow-up after his last visit.

## Discussion

The treatment of male infertility can be discouraging to some patients, due to the inability of physicians to help the patients achieve desirable results. For the past few decades, physicians have learned to understand a variety of etiologies and generate management strategies of azoospermia due to obstruction, causing infertility. One of the rare causes of this obstruction is EDO. While they are mostly harmless, symptoms and conclusions may occur due to their presence causing a disturbance in genitourinary tract physiology. Current literature suggests classifying EDO into two types, congenital and acquired [9]. Based on the patient's history, there was no history of infection, spinal trauma, testicular trauma, or surgery that may cause any traumatic damages to the ejaculatory ducts, suggesting that the EDO might be congenital.

Current studies in the literature utilized a combination of clinical findings, semen analysis, laboratory tests, and imaging modalities. Other studies suggested that vasography is the gold standard imaging modality of EDO, however, in our center vasography could not be performed at the time [10,11]. Therefore, the diagnosis of our patient was made using USG and MRI, later confirmed during urethroscopy. Bilateral hydrocele found during the USG examination was possibly due to the obstruction occurring since puberty. This fact was in accordance with the finding that there was a complaint of inability to ejaculate following an orgasm. TRUS examination showed dilation of the left seminal vesicle and vas deferens with a cystic lesion between them. Based on these results, a pelvic cyst was probable, however, due to the difficulty in evaluating the lesion via USG, the obstruction could not be assessed completely. To determine the type of lesion, an MRI examination was performed. The abdominal MRI results supported the suggestion that there is an obstruction in the right and left ejaculatory ducts or bilateral complete EDO. Urogenital sinus cyst can be classified based on Mayersak's classification [5]. This classification differentiates the midline cysts into two categories, urogenital sinus or ejaculatory duct cyst and Mullerian duct cyst. Ejaculatory duct cyst was determined if spermatozoa were found on cyst aspirate, whereas Mullerian duct cyst had no spermatozoa in the aspirate. An illustration explaining the differences between midline cysts were shown in Figure 6. In our case, MRI examination revealed that there was a high-intensity finding on the T1 Weighted image with no contrast enhancement in the ejaculatory duct orifice. This suggested a high protein content cyst in the ejaculatory duct, speculated to contain spermatozoa. Classic bilateral complete EDO is often presented with low seminal volume, low pH, and azoospermia without fructose in the ejaculate, almost similar to the patient's semen analysis of the specimen [12]. Despite the difficulties of diagnosing EDO, the treatment is simple and effective. The gold standard of EDO is TURED via cystourethroscopy, offering improvement in sperm parameters and symptomatic relief [13]. Approximately 15% of patients with low-volume azoospermia who are treated with TURED eventually develop normal-volume azoospermia. Several retrospective cases have also reported that patients who came and were treated for infertility have a significant increase in the pregnancy rates. Even though the procedure is relatively effective and safe, TURED in young patients should be performed with caution due to the low prostate volume that could potentially lead to increased complications [2].

## Conclusion

As a rare condition, establishing a diagnosis of ejaculatory duct cyst requires precision and accuracy to effectively determine a correct diagnosis and management. TURED is proven to be an effective treatment for ejaculatory duct cysts. Both complete EDO or partial EDO approaches have the same probability of improving semen and sperm quality after TURED. Although the procedure is short, TURED in young patients should be performed with caution due to the low prostate volume that could potentially increase complications.

## Patient consent

Informed consent for patient information to be published in this article was obtained. Appropriate informed consent was obtained for the publication of this case report and accompanying images.

## Ethical approval

This report has been approved by the ethical committee of Dr. Soetomo General-Academic Hospital (Letter of Exemption: 0503/129/I/2021).

## Guarantor

Doddy Moesbadianto Soebadi

## Author contributions

FW, YPK, FR, and DMS contributed equally to this article. All authors have read the manuscript and agreed to the contents.
